# Probiotics in the Management of Vulvovaginal Candidosis

**DOI:** 10.3390/jcm13175163

**Published:** 2024-08-30

**Authors:** Karolina Akinosoglou, Georgios Schinas, Eleni Polyzou, Aristotelis Tsiakalos, Gilbert G. G. Donders

**Affiliations:** 1 Department of Medicine, University of Patras, 26504 Rio, Greece; akin@upatras.gr (K.A.); georg.schinas@gmail.com (G.S.); polyzou.el@gmail.com (E.P.); 2 Department of Internal Medicine and Infectious Diseases, University General Hospital of Patras, 26504 Rio, Greece; 3 Leto General, Maternity & Gynecology Clinic, 11524 Athens, Greece; atsiakalos@gmail.com; 4 Femicare, Clinical Research for Women, 3300 Tienen, Belgium; 5 University Hospital Antwerpen, 2650 Antwerp, Belgium; 6 Department of Obstetrics and Gynecology, Regional Hospital Heilig Hart, 3000 Tienen, Belgium

**Keywords:** vulvovaginal candidosis, recurrent vulvovaginal candidiasis, probiotics, prebiotics, antifungals, vaginosis

## Abstract

Vulvovaginal candidosis (VVC) represents a frequent and cumbersome vaginal infection. Recurrent and/or persistent infections remain common among a significant number of patients despite the use of antifungals. Probiotics offer a promising adjunctive or alternative therapeutic strategy to antifungals in the management of VVC. We aimed to explore and thoroughly examine the various roles and potential applications of probiotics in VVC. A comprehensive literature search was conducted to identify relevant clinical trials and systematic reviews that examine the effectiveness of probiotics in the treatment and prevention of VVC and recurrent VVC (rVVC). Following the initial screening of 4563 articles, a total of 25 clinical studies and seven systematic reviews were finally included in this analysis. The studies reviewed provide a generally positive yet inconsistent view of the efficacy of probiotics in managing VVC, including clinical, mycological response, and prevention perspectives. Nonetheless, fluconazole remains more effective than probiotics in treating VVC, while the combination of the two seems to reduce recurrence and improve symptoms significantly. For prevention, probiotics seem to improve vaginal health and reduce symptoms, while safety and tolerability are consistently reported across the studies, affirming that probiotics represent a low-risk intervention. However, clear conclusions are difficult to establish since relative studies explore different clinical endpoints and follow-up times, variable populations are included, different probiotics are used, and diverse schedules and regimens are administered. We propose that future studies should study the benefit of probiotics in well-defined categories such as (1) treatment with acute probiotics instead of antifungals, (2) adjuvant probiotic therapy together or after antifungals, and (3) VVC recurrence prevention using probiotics.

## 1. Introduction

The concept of using probiotics, defined as «live microorganisms that confer health benefits when consumed in adequate amounts», dates back over a century to the pioneering work of Elie Metchnikoff [1]. Originally focused on improving gastrointestinal health, probiotics have since been explored for their potential benefits in various conditions in both children and adults, e.g., from colic in babies to cardiovascular disease, respiratory infection, and cancer in adults [2]. Various probiotic strains exhibit distinct functions, and specific health benefits for humans have primarily been validated for particular probiotic strains. Probiotics encompass a range of genera, such as *Lactobacillus*, *Bifidobacterium*, *Bacillus*, *Pediococcus*, and various yeasts [2]. Lactobacilli strains [3], in particular, have been thoroughly investigated for their ability to restore and maintain a healthy vaginal microbiome in terms of vaginal pH and bacterial colonization [4,5]. Probiotics designed for vaginal health are readily available in the form of dietary supplements or vaginal capsules/suppositories [6]. When applied vaginally, probiotics act directly at the site of action [7]. In contrast, orally administered probiotics must pass through the gastrointestinal tract before reaching the vaginal tract [8]. Interestingly, research indicates that both routes of administration are effective [9,10]. Oral probiotics, however, may offer additional benefits to vaginal health through the “gut–vagina axis” [8,11]. This involves balancing the gut microbiota [12,13], inhibiting or preventing the migration of urogenital pathogens from the rectum to the vaginal tract [14], and stimulating both gut and systemic immune responses [11,15,16,17].

Their role in the management of bacterial vaginosis (BV) has long been explored [18]. Administered either orally or intravaginally, the proposed mechanisms of action when it comes to BV treatment include competitive exclusion, competition for adhesion sites, production of antimicrobial substances, and modulation of the host immune response [8,19]. A recent meta-analysis of available randomized controlled trials has shown that the cure rate for treating BV with a combination of probiotics and antibiotics was superior to using antibiotics alone [20]. Remarkably, there was no difference in efficacy between probiotics alone and antibiotics alone, while probiotics alone were more effective than a placebo in treating BV [20]. Oral probiotics seemed to be more effective than vaginal administration, and more specifically, oral administration of *L. rhamnosus* [20]. However, one should bear in mind that the effectiveness of probiotic products relies on the number of live cells administered each time. In this context, the ideal probiotic dose remains undefined [21]. It seems that high-dose probiotics were more effective than low-dose probiotics in the management of BV [20]. However, the length of effectiveness varies [20]. A 2019 meta-analysis revealed that probiotics alone were more effective in treating BV in both the short and long term [10]. However, using probiotics after antibiotic treatment was only effective in the short term. The effectiveness varied in short-term follow-up but not in long-term follow-up. Similarly, their role in the prevention of recurrent cases of bacterial vaginosis has also been recently explored and their place is supported in select patients, either with concurrent administration or following antibiotics [5].

The potential role of such probiotics in vulvovaginal candidosis (VVC) remains an open question. VVC represents a common vaginal infection caused by an overgrowth of *Candida* species, primarily *Candida albicans*, but *Candida glabrata*, *Candida krusei*, and other species can also cause symptoms. It manifests with symptoms such as vulvar itching, burning, vulvar redness, excoriation, painful intercourse, and abnormal vaginal discharge, significantly impacting a woman’s quality of life and sexual health. Typically, the initial treatment for acute VVC involves the use of topical or oral azoles [22]. Recurrent and severe cases often require the administration of fluconazole, either as a repetitive single administration or as part of an ongoing suppressive therapy [23,24]. Nevertheless, recurrent and/or persistent infections remain common among a significant number of patients [25]. Additionally, the use of azoles disrupts the normal vaginal flora [26], particularly affecting beneficial fungi such as *Saccharomyces* species, which can play a role in preventing VVC [23,27].

While antifungal medications represent the standard treatment approach, there has been growing interest in exploring alternative and complementary therapies, including probiotics, to address VVC and restore a healthy vaginal microbiome [8]. Firstly, certain probiotics produce enzymes that degrade the biofilm matrix or directly block *Candida* adhesion sites, thereby preventing initial colonization [28]. Secondly, probiotics compete with *Candida* for both adhesion sites on the vaginal epithelium and essential growth nutrients, thereby limiting the resources available to the pathogen [8]. Thirdly, probiotics such as *Lactobacillus* spp. secrete antimicrobial substances, including lactic acid, hydrogen peroxide, and bacteriocins, which directly inhibit both the growth and biofilm formation of *Candida* [29]. Finally, probiotics can modulate the immune response by enhancing the activity of macrophages and dendritic cells and by regulating the balance of pro-inflammatory and anti-inflammatory cytokines, which improves the immunological control over *Candida* proliferation [30].

However, the evidence supporting the efficacy of commercially available probiotics in treating or preventing VVC remains inconclusive and is plagued by significant limitations [31]. While several studies have reported promising results in terms of improved VVC cure and reduced recurrence rates, these findings are often limited by methodological flaws, such as small sample sizes, the lack of rigorous study designs, and a high risk of bias. It is noteworthy that the majority of the studies evaluated in the systematic review by van de Wijgert and Verwijs [31] were considered to have a high overall risk of bias, with only one randomized controlled trial showing a medium risk [31,32]. This latter trial found no significant difference in VVC incidence between probiotic and placebo users, urging the authors to call for more robust evidence to establish the true efficacy of probiotics in VVC management [32]. Prebiotics, such as oligosaccharides and polysaccharides, have also garnered interest as potential alternatives or adjuncts to probiotics in the management of vaginal infections, including VVC [33]. These compounds aim to selectively stimulate the growth of beneficial lactobacilli while inhibiting the proliferation of pathogens, such as *Candida* species. However, the evidence in the area of yeast infections is even more limited than with bacterial infections in the vagina, with only a few clinical studies evaluating the efficacy of prebiotics in VVC management.

To comprehensively address the complexity of the subject, this review aims to explore the various roles and potential applications of probiotics in VVC. An extensive literature review has been conducted, adopting a rigorous methodology akin to that used in systematic reviews. From this review, data pertinent to the impact of probiotics on VVC have been meticulously extracted and systematically organized into three categories: the first concerns the treatment of VVC with probiotics; the second focuses on the preventive applications of probiotics, while the third compiles all evidence available from the systematic reviews pertaining to the use of probiotics in managing VVC. This structured approach aims to provide a clear and comprehensive synthesis of the current evidence, identifying both the potential benefits and limitations of probiotics in VVC management, thereby informing future research directions and clinical practices.

## 2. Materials and Methods

A comprehensive literature search was conducted using PubMed, Science Direct, and the Cochrane Database to identify relevant clinical trials and systematic reviews that examine the effectiveness of probiotics in the treatment and prevention of VVC and recurrent VVC (rVVC). The search was formulated using a combination of terms related to probiotics, including specific probiotic strains, and terms associated with VVC and rVVC. The search string employed was: (“probiotics”, “probiotic therapy”, “probiotic supplementation”, “*Lactobacillus rhamnosus*”, “*Lactobacillus reuteri* “,”*Lactobacillus acidophilus*”, “*Lactobacillus gasseri*”, “*Lactobacillus fermentum*”, “*Lactobacillus plantarum*”, “*Lactobacillus*”, “*Bifidobacterium bifidum*”, “*Bifidobacterium lactis*”, “*Bifidobacteria*”, “*Saccharomyces cerevisiae*”, “*Saccharomyces boulardii*”, “microbial supplements”) AND (“vaginal candidiasis”, “vulvovaginal candidiasis”, “vaginal yeast infection”, “candida vaginitis”, “vaginal candidosis”, “vulvovaginal candidosis”, “yeast vaginitis”, “*Candida albicans*”, “vaginal *Candida*”, “VVC”, “candidiasis, vulvovaginal” [MeSH Terms]). The search was limited to articles published in peer-reviewed journals.

### 2.1. Study Selection and Data Extraction

Four independent reviewers performed a literature search and identified articles, which were then hand-searched for relative references. Articles were included if they were clinical studies or systematic reviews that reported on the effectiveness of probiotics in treating or preventing VVC or rVVC. Studies that reported outcomes related to bacterial vaginosis were also included if they contained relevant data on VVC outcomes or mentioned VVC as an adverse event. Additionally, the relevant literature was identified through the targeted screening of references and related articles on PubMed. Non-English publications, non-human studies, and non-systematic reviews were excluded from consideration. Disagreements were discussed and resolved.

**Figure 1:**
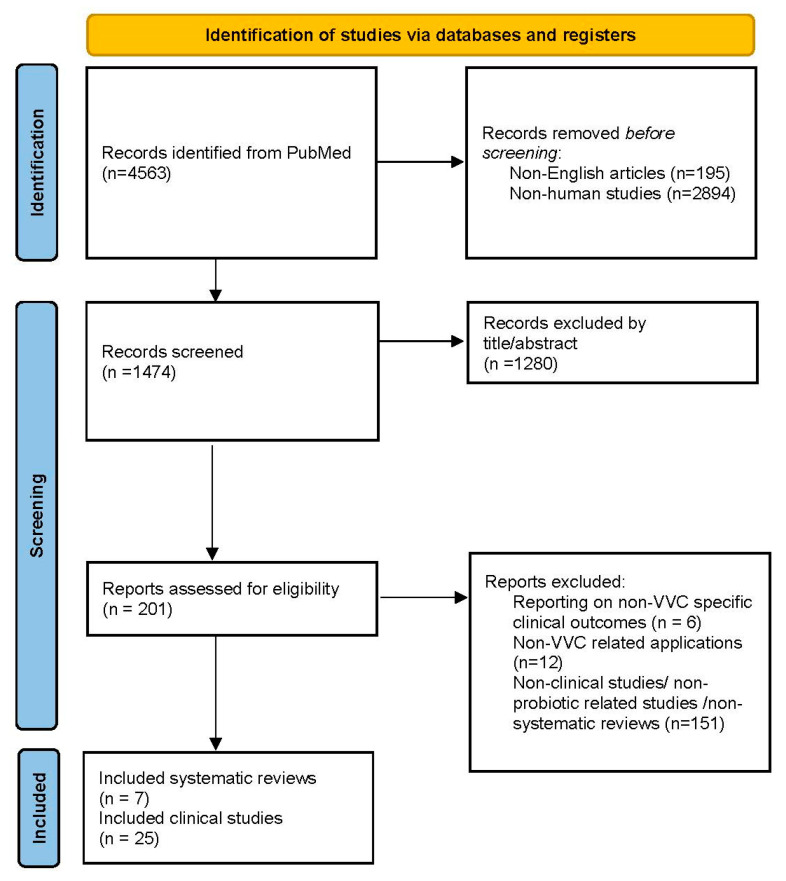
The selection process is visualized using a PRISMA flowchart, which delineates the stages of article elimination and selection.

The selection process was visualized using a PRISMA flowchart (Figure 1), which delineated the stages of article elimination and selection. Data were systematically extracted and categorized into four tables: (1) clinical trials concerning the treatment of VVC/rVVC (Table 1), (2) clinical trials concerning the prevention of VVC/rVVC (Table 2), (3) clinical trials investigating the effectiveness in special populations (Table 3), and (4) systematic reviews focusing on VVC-specific outcomes following probiotic administration (Table 4).

### 2.2. Data Synthesis

Data from the included studies were synthesized narratively. The synthesis focused on the diversity of probiotic strains used, dosages, treatment durations, and patient populations. Efficacy outcomes, including rates of symptom resolution, recurrence prevention, and incidence of adverse events, were synthesized. Considering the heterogeneity of the identified studies, the narrative synthesis allows for a comprehensive understanding of the variability and efficacy of probiotic interventions across different study designs and population subsets, facilitating an in-depth investigation of their therapeutic potential and limitations.

## 3. Results

Briefly, we found 17 clinical studies investigating the effectiveness of probiotics in treating VVC and/or rVVC (Table 1 and Table 3) [34,35,36,37,38,39,40,41,42,43,44,45,46,47,48,49,50], eight clinical studies investigating the effectiveness of probiotics in preventing VVC and/or rVVC (Table 2) [51,52,53,54,55,56,57,58], and seven Systematic Reviews reporting on VVC-specific outcomes following probiotic administration (Table 4) (seven Systematic Reviews, 0 Metanalysis) [31,59,60,61,62,63,64].

In terms of the effectiveness of probiotics in treating VVC or rVVC, the effect of probiotics vs. antifungals was examined in two studies (n = 2) [35,42]. Fluconazole showed better long-term outcomes in one study while higher patient satisfaction was observed with fluconazole treatment [42]. Similar short-term efficacy in symptom reduction and safety profiles were noted in both studies [35,42]. In this context, probiotics were compared to a placebo in ten studies (n = 10) [36,37,38,40,44,46,47,50]. Probiotics reduced symptoms such as discharge and itching in five studies [37,38,40,44,46]. Increased *Lactobacillus* counts in vaginal flora were observed in four studies [38,40,47,50]. Lower recurrence rates and higher cure rates were reported in six studies [36,37,44,46,47,50].

Probiotics were studied as an adjunct therapy for VVC, examining the synergy with azoles in five studies (n = 5) [39,41,43,45,49]. Combined probiotic and antifungal treatments showed higher cure rates and lower recurrence rates compared to antifungal treatment alone in several studies. Probiotics enhanced the efficacy of antifungal treatments in reducing symptoms and restoring vaginal flora [39,49]. Significant improvements in patient satisfaction were observed with combined treatments [49].

Probiotics were used for the prophylaxis of VVC and compared with placebos in six studies (n = 6) [52,53,54,56,57,58]. Probiotics improved vaginal health and reduced symptoms in four studies [53,54,57,58]. Decreased recurrence rates of VVC were observed in one study [54]. Probiotics were well-tolerated, with no significant adverse effects reported in five studies [52,53,54,57,58]. No significant difference was found in preventing post-antibiotic vulvovaginitis in one study [56]. In the same context, probiotics and antifungals vs. antifungals alone were tested in two studies (n = 2) [51,55]. The combination therapy increased *Lactobacillus* levels and improved symptoms [51,55]. Significantly reduced recurrence rates and better long-term outcomes were reported [55]. High compliance and good tolerability were observed [51,55].

Systematic reviews on the efficacy of probiotics in treating VVC or rVVC have shown that probiotics with antifungal drugs improve short-term cure rates, resulting in a reduction in relapse rates within one month and showing cure rates between 57–100% [31,59,60,61,62,63,64]. However, mixed results on long-term cures were found, even though good tolerability and safety was reported across all studies. Of note, a high risk of bias was recorded in all studies, while in two, no quality assessment was reported [59,63].

Moreover, only a limited number of studies explored the alterations of microbiota in the vaginal mucosa during VVC and the role of probiotics in their restoration [38,39,40,47,50], while various bacteria were used in each study, including *L. acidophilus* [38,42,53,63], *L. crispatus* [40], *L. plantarum* [35,51,55,58], or a mix of more than two [34,36,37,38,39,41,42,43,44,45,47,54,56,57]. Routes of application were diverse, including vaginal [31,35,37,38,39,48,49,50,51,54,55], oral [34,36,42,46,48,53,56,57], or both [40,45,59]. The preferred concentration of bacteria in the different clinical studies was mostly >10^9^ CFU.

**Table 1:** Clinical Trials investigating the effectiveness of probiotics in treating VVC and/or rVVC.

Citation	Year	Country	Study Design	Study Population	Intervention	Control	Results/Outcomes
Bertarello C et al. [35]	2024	Italy	Randomized, investigator-blinded, active-controlled, multicenter, two-parallel-group study	Premenopausal women aged 18–45 with VVC	*L.plantarum* P17630 100,000,000 CFU soft vaginal capsules (LJ LACTO) for 3 consecutive days	Miconazole nitrate 400 mg soft vaginal capsules (DAKTARIN^®^) for 3 consecutive days	- Similar efficacy in reducing symptoms of VVC without significant difference between groups (*p* > 0.05 for each symptom, at each time point). - No significant difference in the change of mean VAS scores for any symptom from baseline to visit 2 in patients considered healed and from baseline to visit 3 in those who continued the treatment for 6 days (*p* > 0.05). - The mean concentration of IL-6 decreased from visit 1 to visit 3 in both groups without a significant difference (*p* > 0.05) between groups. - No severe or non-serious adverse events were reported in both groups.
Mändar R et al. [40]	2023	Estonia	Randomized double-blind placebo-controlled two-arm parallel trial.	182 women recruited with vaginitis—89 BV and 93 VVC (aged 18–50 years)	Probiotic capsules (either oral or vaginal probiotic capsules, administered over three months) containing *L. crispatus* strains—DSM32720, DSM32718, and DSM32716, in case of VVC-. (3 × 10^10^ CFU/capsule).	Capsules containing maltodextrin	- Clinical Improvements in VVC: Symptom Relief: Both (oral or vaginal) probiotic groups experienced significant reductions in the amount of discharge and itching. Vaginal Probiotic Group: significant relief from itching (*p* = 0.035) and discharge (*p* = 0.047) Oral Probiotic Group: Statistically significant reduction in discharge (*p* = 0.050) - Microbial Analysis: *Lactobacilli* Increase: statistically significant increase in *Lactobacillus* counts in the vaginal samples from the vaginal probiotic group post-treatment (*p* = 0.028). Other Microorganisms: *G. vaginalis* Counts: Decreases were more pronounced in the oral probiotic group, although not statistically significant (*p* = 0.114). - Safety and Tolerability: No serious adverse events were reported across both treatment groups.
Mollazadeh-Narestan Z, et al. [42]	2023	Iran	Triple-blinded, placebo-controlled RCT	80 married women, 18–49 years old, with VVC as confirmed by clinical and laboratory diagnosis	1 probiotic capsule p.o. per day for 30 days containing 10^9^ CFU/g LA-5 (*L. acidophilus*-5) plus a single placebo fluconazole capsule	Single dose of oral fluconazole (150 mg) + 1 probiotic placebo capsule per day for 30 days	- No significant difference in negative culture at 30–35 days (*p* = 0.127). - The control group showed a significantly higher frequency of negative culture at 60–65 days (*p* = 0.016). - No statistically significant difference in vaginal pH - Less abnormal discharge and vulvovaginal erythema in the control group in the first and second follow-ups (*p* < 0.05). - No significant difference in burning, frequent urination, dysuria, and dyspareunia (*p* > 0.05). - Higher satisfaction with fluconazole treatment (*p* = 0.020)
Zeng X et al. [50].	2023	China	Prospective observational study	42 women, aged from 20–50 years old with VVC	Single 500 mg dose of clotrimazole vaginal tablet plus a 7-day course of Lacidophilin vaginal capsules (2 tablets per day, 1.20 × 10^7^ CFU/day)	Healthy control group with normal vaginal flora	Treatment efficacy: 46.67% of VVC patients cured at second visit, 61.54% at third visit, eventually 72.73% in per-protocol population Microbiome diversity: After treatment, bacterial diversity of VVC patients increased gradually. Significant increase in Shannon index, indicating recovery of vaginal microbes Microbiome composition: Dominant genus in both groups was Lactobacillus, abundance of vaginal flora increased in VVC patients over course of treatment
Vahedpoor Z, et al. [45]	2021	Iran	Randomized, double-blind, placebo-controlled clinical trial	40 women with rVVC or BV, aged 18–48 years	Fluconazole 150 mg + vaginal and oral probiotics (*L. acidophilus, L. plantarum, L. rhamnosus, L. gasseri*) for 14 nights and 30 days.	Fluconazole 150 mg + vaginal and oral placebo for 14 nights and 30 days	Significant reduction in vaginal discharge and positive culture in the probiotic group compared to the placebo group (10.3% vs. 35.6%, *p* = 0.03; 10.3% vs. 38.5%, *p* = 0.014). Significant improvements in itching, discharge, and pH (*p* < 0.05). Significant improvement in burning, discharge, and itching with probiotics (*p* ≤ 0.05, ORs = 6.21, 7.38, 13.82, respectively). No significant difference in mycological cure between groups (*p* = 0.184); nonetheless, this was high (68.4%) in the probiotic group. No significant difference in fluconazole susceptibility (*p* = 0.181).
Donders G et al. [37]	2020	Belgium	Prospective proof-of-concept study	- 20 pre-menopausal, Caucasian women with acute VVC, aged 18–50 years, with positive *Candida* microscopy and/or culture, and specific signs or symptoms	Probiotic gel containing *L. plantarum* YUN-V2.0, *L. pentosus* YUN-V1.0, and *L. rhamnosus* YUN-S1.0. (2.5 mL of gel administered intra-vaginally during 10 consecutive days, containing 10^9^–10^10^ CFU of lactobacilli per gram of gel.)	None	Cure Rate: 45% of participants achieved a cure with the probiotic gel alone. Rescue Medication: 45% completed the study without the need for any rescue medication. Those requiring rescue treatment had experienced twice as many Candida infections historically. Symptoms: Rapid improvement in symptoms in women who did not need rescue medication (*p* = 0.009). Tolerability and Acceptability: No notable side effects were reported; 74% of participants found the gel comfortable to use and 42% would use the gel again for this indication. Severity: Women with severe symptoms often required immediate antifungal treatment, whereas the gel showed potential as an adjuvant therapy or as a standalone treatment in mild to moderate cases.
Russo R et al. [44]	2019	Romania	Double-blinded RCT	48 pre-menopausal women aged 18–50 years with symptomatic acute VVC episodes, positive cultures for *Candida* spp., and documented history of recurrences	Respecta^®^, containing a proprietary *Lactobacilli* mixture (5 × 10^9^ CFU per capsule) including *L. acidophilus* GLA-14 and *L. rhamnosus* HN001 in combination with bovine lactoferrin RCX™ (50 mg). Treatment consisted of an induction phase with standard antifungal therapy (clotrimazole 100 mg daily for 7 days) and simultaneous administration of Respecta^®^ or placebo (2 capsules/day for 5 days followed by 1 capsule/day for 10 days) followed by a maintenance phase (1 capsule/day for 10 days per month in the premenstrual phase) for 6 months.	Placebo of maltodextrin (100 mg) with the same excipients as Respecta^®^	- Clinical cure rates: significantly higher in the Respecta^®^ group for itching and vaginal discharge at 3- and 6-month follow-ups (T4 and T5) (*p* < 0.01). - Overall cure rates: significantly higher in the Respecta^®^ group at T4 and T5 (*p* < 0.01). - Recurrence rates: significantly lower in the Respecta^®^ group at T4 and T5 (*p* < 0.01).
Davar R et al. [36]	2016	Iran	Double-blinded RCT	- Total of 64 included in the study - 59 patients studied (31 placebo, 28 probiotic) - All women had clinical signs and symptoms of VVC, excluding diabetic, pregnant, postmenopausal, and menstruating women	Pro-Digest, twice a day for 10 days orally, containing: - *L.* acidophilus [7:5 ×10^9^ CFU/Cap] - *Bifidobacterium bifidum* [6 × 10^9^ CFU/Cap] - *Bifidobacterium longum* [1:5 × 10^9^ CFU/Cap]	Oral placebo capsules containing no effective substances	Recurrence rates over 6 months: - Probiotic group: 11 cases (35.5%) - Placebo group: 2 cases (7.2%) - *p* = 0.01, OR 0.14, 95% CI (0.028–0.7)
Pendharkar S, et al. [43]	2015	Sweden	2 pilot open-label clinical trials Trial II concerning rVVC	40 women diagnosed with BV or rVVC based on Amsel’s criteria or clinical symptoms. Trial II included women with rVVC (9 women in treatment arm and 10 women on placebo)	28-day course of fluconazole (50 mg qd orally), with vaginal EcoVag^®^ (*L. rhamnosus* DSM14870 and *L. gasseri* DSM14869 from days 18 to 28, then post-menstruation for 10 days, and fluconazole 200 mg weekly for two months, followed by bi-weekly doses of 200 mg for three months and weekly EcoVag^®^ for four months.	Extended fluconazole treatment	rVVC in Trial II: Women treated with fluconazole and EcoVag^®^ had cure rates of 100% at 6 months and 89% at 12 months. In contrast, those treated with only fluconazole had cure rates of 100% at 6 months and 70% at 12 months.
Kovachev SM et al. [39]	2015	Bulgaria	Single-center, open-label RCT	436 women aged 17–50 with *C. albicans* vaginal infections	Single oral dose of fluconazole (150 mg) + single vaginal globule of fenticonazole (600 mg), followed by ten doses of vaginal probiotic containing *L. acidophilus, L. rhamnosus, S. thermophilus, L. delbrueckii* subsp. *Bulgaricus*, starting on the 5th day of azole treatment(Group 2)	Single oral dose of fluconazole (150 mg) + single vaginal globule of fenticonazole (600 mg) (Group 1)	Clinical Complaints Improvement: Group 2 doubled the improvement in clinical complaints compared to Group 1 (Group 1: 15.38%, *p* = 0.001; Group 2: 29.06%, *p* = 0.005). Vaginal Tissue Changes: Higher percentage of improvements in vaginal tissue changes in Group 2, although not statistically significant (Group 1: 72.66%, *p* = 0.387; Group 2: 94.61%, *p* = 0.594). Vaginal Fluorine Levels: Both groups showed great improvement (Group 1: 94.44%, *p* = 0.031; Group 2: 98.55%, *p* = 0.308). Vaginal pH Reduction: All participants in both groups achieved a reduction in alkaline pH, with similar effectiveness (Group 1: 97.73%, *p* = 0.098; Group 2: 98.66%, *p* = 0.757). Native Microscope Slide Observations: Group 2 demonstrated significantly higher improvement in spores and filaments (Group 1: 67.96%, *p* = 0.17; Group 2: 94.17%, *p* = 0.67). *Lactobacilli* Dominance Increase: Group 2 showed a more pronounced increase in *Lactobacilli* dominance, achieving nearly complete prevalence (Group 1: 91.79%, *p* = 0.02; Group 2: 98.6%, *p* < 0.01). Culture Testing for *C. albicans*: There was a more significant reduction in *C. albicans* presence in Group 2, (Group 1: reduction to 36.7%; Group 2: reduction to 4.8%, *p* = 0.82).
Vujic G et al. [47]	2013	Croatia	Randomized, double-blind, multicentric, placebo-controlled trial	544 women > 18 years with vaginal infection (BV, VVC, trichomoniasis, or a combination)	“Lactogyn” containing >10^9^ CFU *L. rhamnosus* GR-1 and *L. reuteri* RC-14, two capsules per day for 6 weeks orally	Identical looking placebo capsules, two per day for 6 weeks	Primary outcome: Restitution of normal vaginal microbiota after 6 weeks—Placebo group: 26.85% achieved restitution vs. probiotic group: 61.52%, *p* < 0.001. NNT: 2.9, RRR: 47.4%. Rate of recurrence of vaginosis after additional 6 weeks: Placebo group: 20.81% vs. probiotic group: 51.14%, *p* < 0.001. NNT: 3.3, RRR: 38.3%. Even though % of patients with fungal vaginosis decreased in the probiotic group, the results did not clearly distinguish a statistical significance between VVC and BV. *Lactobacillus* recovery: Higher in probiotic group (81.5%) compared to placebo group (28.9%), *p* < 0.001. Demographic variables not significantly related to outcomes except for number of children (*p* = 0.082). Women with 3 or more deliveries are less likely to achieve restitution compared to those with 2 or fewer deliveries.
Vicariotto F et al. [46]	2012	Italy	Pilot trial	30 women with VVC, aged between 23 and 64 years	ActiCand 30: *L. fermentum* LF10 and *L. acidophilus* LA02 in slow-release effervescent vaginal tablets	None	86.6% resolution of *Candida* symptoms after 28 days (*p* < 0.001) Low recurrence rate of 11.5% at 56 days (*p* = 0.083)
Ehrström S. et al. [38]	2010	Sweden	Double-blind, placebo-controlled, parallel design	95 women aged 18–45 diagnosed and treated for VVC and/or BV	Vaginal capsules containing a mixture of freeze-dried *L. gasseri* LN40, *L. fermentum* LN99, *L. casei* subsp. *rhamnosus* LN113, and *P. acidilactici* LN23	Identical placebo capsules containing only the carrying matrix	- Colonization: 89% of the intervention group vs. 0% of the placebo group had one or more LN strains present in the vagina after supplementation (*p* < 0.0001). - Clinical Outcome: No significant difference in clinical cure rates between groups. Intervention group reported less malodorous discharge post-supplementation (*p* = 0.03) and after the second menstruation (*p* = 0.04), compared with placebo. - Adverse Events: Few adverse events, no serious adverse events recorded.
Witt A et al. [49]	2009	Austria	Single-centre, prospective, randomized trial.	150 women with a history of RVVC and an acute episode of VVC	- Itraconazole and lactobacilli group: Received the same regimen as the itraconazole group, plus one tablet of vaginal lactobacilli to be taken every evening for six consecutive days. [Group 2 (G2)]	- Itraconazole group: Induction regimen of a single-day 200 mg bid itraconazole, followed by maintenance of 200 mg bid once a month for 6 months. [Group 1 (G1)] - Classic homeopathy group: High potencies of a single homeopathic remedy for the 12-month study period. [Group 3 (G3)]	Culture-Free Status Achievement: Itraconazole (Group 1) and itraconazole plus lactobacilli (Group 2) groups achieved a culture-free status significantly earlier than the classic homeopathy group (Group 3) (*p* < 0.0001). At the start of the maintenance regimen, 89.8% of women in G1, 85% in G2, and 47% in G3 were free of detectable cultures. Recurrence Rates: G3 experienced recurrences of vaginal Candida infection significantly earlier compared to those in G 1 and 2 (*p* = 0.002). After 12 months, 76% of G1, 78% of G2, and 39% of G3 remained culture-free. End of Study Candida-Free Status: At the end of the study, a higher percentage of G1 and G2 were free of culture-detectable Candida compared to G3. Discomfort Levels and Treatment Satisfaction: G3 reported higher discomfort levels and lower satisfaction with treatment compared to G1 and G2 VAS score for G3 was 36.8, significantly higher than 25.1 in G1 and 27.7 in G2 (*p* < 0.001). Satisfaction scores were lower in G3 (59.2%) compared to 68.2 in G1 and 71.7 in G2 (*p* < 0.001).
Martinez RC et al. [41]	2009	Brazil	RCT	68 patients with VVC	Single dose of Fluconazole (150 mg) plus Probiotic capsules containing *L. rhamnosus* GR-1 and *L. reuteri* RC-14, 1 × 10^9^ viable cells each, daily for 28 days	Single dose of Fluconazole (150 mg) plus Placebo capsules containing cellulose and magnesium stearate	- Subjects treated with fluconazole and probiotics showed a higher cure rate of VVC compared to those treated with fluconazole and placebo (*p* < 0.05). - Subjects with a history of recurrent VVC showed fewer yeast isolates in the probiotic group compared to the placebo group (*p* < 0.05). - Clinical cure rates did not significantly differ between the two groups (*p* > 0.05).

VVC: Vulvovaginal Candidosis; rVVC: recurrent VVC; RCT: Randomized controlled Trial; p.o.: by mouth; CFU: colony-forming units; IL-6: interleukin 6; NNT: number needed to treat; RRR: relative risk ratio; OR: odds ratio; BV: Bacterial Vaginosis; VAS: Visual Analogue Scale.

**Table 2:** Clinical Trials investigating the effectiveness of probiotics in preventing Vulvovaginal Candidiasis (VVC) and/or Recurrent-VVC.

Citation	Year	Country	Study Design	Study Population	Intervention	Control	Results/Outcomes
Vladareanu R et al. [58]	2018	Romania	Multicenter, placebo-controlled RCT	93 Caucasian women >18 years, with a clinical history of recurrent yeast vaginitis (>3 relapses within 1 year)	*L. plantarum* P17630 (5 × 10^9^ CFU/capsule) administrated by oral capsule daily in 3 cycles of treatment, each consisting of 15 days intake and 15 days washout (Overall Duration: 90 days)	Placebo	Significant increase in - Participants with LBG I score after the first treatment cycle (*p* = 0.000016) - Percentage of women with LBG I score remained significantly higher than baseline value after three cycles of treatment at day 90 (*p* = 0.001415) - *L. plantarum* P17630 more efficient than placebo in reducing vaginal mucosa redness at day 45 (*p* = 0.006) and day 90 (*p* = 0.001) - *L. plantarum* P17630 significantly decreased the intensity of swelling at day 45 (*p* = 0.002) and day 90 (*p* = 0.001) - Treatment well tolerated; no adverse effects recorded throughout the study
Palacios S, et al. [55]	2016	Spain	Open-label, prospective, non-randomized	Sexually active women 18–50 with symptomatic acute VVC	Single-dose 500 mg vaginal tablet of clotrimazole followed by vaginal tablets with 10^8^ CFU *L. plantarum* I1001 as adjuvant therapy	Clotrimazole 500 mg only	Probiotic group had higher recurrence-free survival at 3 months (72.83% vs. 34.88%, HR: 0.30, 95% CI: 0.10–0.91, *p* = 0.033) and 59% reduction in VVC episodes (*p* = 0.001). High compliance (91.3%). Higher recurrence with prior antibiotic use (HR: 10.46, 95% CI: 2.18–50.12, *p* = 0.003). Positive effect observed at 6 months in women with rVVC.
Murina F et al. [54]	2014	Italy	Observational study	58 patients with acute symptomatic VVC, confirmed by microscopy, with a history of rVVC (≥ 4 culture-confirmed episodes in a 12-month period) Age range: 21 to 47 years	Oral fluconazole 200 mg orally for 3 alternate days during the first treatment week; Vaginal tablets containing *L. fermentum* LF10 and *L. acidophilus* LA02 (at least 0.4 billion live cells each), arabinogalactan, fructooligosaccharides, citric acid, and sodium bicarbonate. One tablet inserted into the vagina on alternate days for 10 consecutive nights, then 1 tablet weekly for the subsequent 10 weeks	None	During the 10-week prophylactic phase, 86.0% of patients remained without clinical recurrence. During the 7-month observation period after treatment cessation, 42 patients of 49 (85.7%) were symptom-free at the end of the protocol, whereas clinical recurrences occurred in 7 women (14.3%). 72.4% of patients experienced no clinical recurrence throughout the 7-month observation phase
De Seta F et al. [51]	2014	Italy	Retrospective comparative study	89 women with acute VVC	Group B: Clotrimazole vaginal cream + vaginal lubricant + vaginal application of capsule containing *L. plantarum* P17630 (>10^8^ CFU) once a day for 6 days and then once a week for another 4 weeks beginning the day following clotrimazole discontinuation	Group A: Clotrimazole vaginal cream + vaginal lubricant	- Group A: Non-significant increase in recurrence (12.5% versus 2.5%, *p* = 0.095) and significant increase in pH > 5 (40% versus 0%, *p* < 0.01) - Group B: Significant increase in *Lactobacillus* values (80% versus 40%, *p* < 0.001) and subjective improvement in symptoms reported by 90% of patients (*p* = 0.03)
Hemmerling A et al. [52].	2009	USA	Phase 1, placebo-controlled, dose-ranging, randomized, double-blind	12 sexually abstinent women 18–40 years, divided into three blocks of 4 participants each, with dosages set at 5 × 10^10^, 1 × 10^10^, and 2 × 10^10^ cfu/dose. The randomization was in a 3:1 ratio of active product to placebo.	Prefilled vaginal applicators containing 3 doses (5 × 10^8^, 1 × 10^9^, and 2 × 10^9^ CFU) of LACTIN-V, applied vaginally once daily for 5 consecutive days	Placebo applicators, applied vaginally once daily for 5 consecutive days	Tolerability: All participants completed the study without discontinuing due to adverse events (AEs). Exposure of the LACTIN-V and placebo applicators to vaginal mucus was high, with 93% for LACTIN-V and 100% for placebo. Acceptability: The majority of participants (83%) expressed satisfaction and comfort with the study product, agreeing or strongly agreeing with related statements. Safety and Adverse Events: A total of 45 adverse events: 69% (31 events) were genitourinary (GU)-related. [vaginal discharge (42%), abdominal pain, metrorrhagia, vulvovaginitis (each affecting 33% of subjects), and vaginal candidiasis and vaginal odor (each affecting 25% of subjects). The severity of AEs was predominantly mild, with 91% (41 events) classified as grade 1. All four moderate AEs (grade 2) were determined to be unrelated to product use. There were no grade 3 or 4 severity AEs or serious adverse events (SAEs). Laboratory parameters and colposcopy findings remained within normal limits or were clinically insignificant
Pirotta M et al. [56]	2004	Australia	2 × 2 factorial, RCT	278 women, 18–50 with non-gynecological infections	Oral Powder: Lactobac (*L. rhamnosus* and *Bifidobacterium longum*) or placebo, half a teaspoon twice daily 20 min before meals for 10 days. Vaginal Pessary: Femilac (*L. rhamnosus*, *L. delbrueckii*, *L acidophilus*, and *Streptococcus thermophilus*) or placebo, one pessary at night for 10 days.	Placebo for both oral and vaginal interventions	Overall incidence of post-antibiotic vulvovaginitis was 23%. No significant difference observed in the development of vulvovaginitis between intervention and placebo groups: Oral lactobacillus: OR 1.06, *p* = 0.814 (95% CI 0.58 to 1.94); Vaginal lactobacillus: OR 1.38, *p* = 0.347 (95% CI 0.75 to 2.54).
Reid, G. et al. [57]	2001	Canada	Observational Study	10 women; asymptomatic for infection but with a recent history of urogenital infection; some had recurrent yeast vaginitis, BV, and UTIs	Probiotic solution containing >10^9^ CFU viable *L. rhamnosus* GR-1 and *L. fermentum* RC-14, administered orally each morning and last thing at night for 14 days.	None	- *L. rhamnosus* GR-1 and/or *L. fermentum* RC-14 recovered from the vagina within one week in all patients. - Improved well-being reported by all patients. - Eradication of enterococci from the bladder and vagina in one patient. - No side effects reported. - No recurrent yeast vaginitis, BV, or UTIs during treatment and follow-up (up to 12 weeks).
Hilton E, et al. [53]	1992	USA	Crossover trial	33 women with rVVC; 21 eligible after exclusions, 13 completed the protocol	Daily ingestion of yogurt containing >10^8^ CFU viable *L. acidophilus* for 6 months	Yogurt-free diet for 6 months	Significant decrease in candidal infections and colonization when consuming yogurt. Mean number of infections per 6 months: Control—2.54 +/− 1.66, Yogurt—0.38 +/− 0.51 (*p* = 0.001). Candidal colonization: Control—3.23 +/− 2.17 per 6 months, Yogurt—0.84 +/− 0.90 per 6 months (*p* = 0.001).

VVC: Vulvovaginal Candidosis; rVVC: recurrent VVC; BV: Bacterial Vaginosis; UTI: Urinary tract Infection; CFU: colony-forming units; RCT: Randomized controlled Trial; p.o.: by mouth; HR: hazard ratio; CI: confidence interval.

**Table 3:** Clinical Trials investigating the effectiveness of probiotics in treating and preventing VVC in special populations.

Citation	Year	Country	Study Design	Study Population	Intervention	Control	Results/Outcomes
Ang X-Y et al., [34]	2022	Malaysia	Double-blind, placebo controlled RCT	78 pregnant women at 14–32 weeks of pregnancy with confirmed VVC (lactobacilli group, n = 39; placebo group, n = 39).	Oral administration of two capsules/day of lactobacilli (SynForU-HerCare) containing a mixture of *L. plantarum* LP115, *L. helveticus* LA25, *L. rhamnosus* LRH10, *L. paracasei* LPC12, *L. fermentum* LF26, and *L. delbrueckii* subsp. lactis LDL114 at a dosage of 9.5 log CFU/capsule, with maltodextrin as an excipient, for 8 weeks.	Capsules containing 100% maltodextrin for the same duration.	- Vulvovaginal Symptoms: - Patients on lactobacilli showed reduced symptoms of irritation (*p* = 0.023), discharge (*p* = 0.011), and burning (*p* = 0.046) after 8 weeks. - Itching symptoms were reduced in all patients since week 4. - Emotional and Social Symptoms: - Patients on lactobacilli showed reduced emotional stress and social impacts related to vulvovaginal symptoms after 8 weeks. - Recurrences of Symptoms: - Patients on lactobacilli showed reduced recurrences of emotional stress and social impacts compared to the placebo group at both week 4 and week 8. - Gut Health: - Patients on lactobacilli showed higher defecation times per week at week 4 (*p* = 0.010) and week 8 (*p* = 0.001) compared to the placebo group.
Williams AB et al. [48]	2001	United States	Double-blind, placebo-controlled RCT	164 women > 18 years of age, HIV-positive, not currently using antifungal medications, not pregnant, and free of VVC at enrollment.	Capsules containing clotrimazole powder 100 mg or *L. acidophilus*, which they were instructed to insert vaginally	Placebo capsules	Incidence of VVC: Over an average follow-up of 19–22 months: Clotrimazole group: 7 cases of VVC occurred. *L. acidophilus* group: 9 cases occurred. Placebo group: 18 cases occurred, including two women with recurrent episodes. - Treatment Effects: Clotrimazole: Reduced the risk of VVC, with a RR of 0.4 (95% CI = 0.2, 0.9; *p* = 0.03 compared to placebo). *L. acidophilus*: Showed a potential reduction in VVC risk, with a RR of 0.5 (95% CI = 0.2, 1.1; *p* = 0.09 compared to placebo),

VVC: Vulvovaginal Candidosis; RCT: Randomized Clinical Trial; CFU: Colony-Forming Units; RR: relative risk; CI: Confidence Interval; HIV: Human Immunodeficiency Virus.

**Table 4:** Systematic Reviews reporting on VVC-specific outcomes following probiotic administration.

Citation	Year	Number of Studies Included	Included Studies Design	Participants	Intervention/Comparison	Results/Findings	Quality Assessment	Limitations
Van de Wijgert et al. [31]	2020	12 studies reporting on VVC outcomes (Countries of origin not reported)	RCTs: 4 Pre/Post-Intervention Studies: 6 Prospective Cohort Studies: 2	Sexually active women (total number not specified)	Various vaginally applied probiotics vs. Placebo or No intervention/with or without additional oral or vaginal antibiotic or antifungal treatment	- Four studies reported VVC cure rates ranging from 57% to 100% after treatment durations of 6 days to 2 months without antifungal use. However, long-term recurrence post-treatment cessation was not evaluated in these studies. - Eight studies examined VVC recurrence or incidence. Two studies comparing antifungal plus probiotic use with antifungal use alone found no difference in recurrence. Conversely, two studies comparing probiotic after antifungal use with either placebo or antifungal alone showed significantly lower recurrence rates. One study found a reduced VVC incidence after antibiotic use for non-VVC indications, while another study in women using vaginal probiotics without antifungal or antibiotic therapy showed similar VVC incidence compared to placebo users.	High risk of bias: 11 studies Medium risk of bias: 1 study	Suboptimal quality of most studies
Jeng HS et al. [61].	2020	9 studies specifically on VVC	RCTs and two-armed prospective studies	1220 patients evaluated for VVC	Probiotics vs. placebo in the treatment of VVC	VVC-specific findings: Significant reduction in recurrence rate at 1-month post-treatment in the probiotic group compared to control (pooled OR = 0.27, 95% CI: 0.16–0.45, *p* < 0.001). Favorable outcomes observed at 1 month with a pooled OR = 1.72, 95% CI: 1.13–2.64, *p* = 0.012 for cure or remission rates.	High heterogeneity in some results (I2 = 27% for VVC at 1-month recurrence).	High heterogeneity among studies; lack of long-term follow-up data for some outcomes
Xie et al. [64]	2017	10: China (7), Brazil (1), Bulgaria (1), and Iran (1)	RCTs	1656 participants, non-pregnant women with VVC	Probiotics as adjuvants to conventional antifungal drugs vs. conventional antifungal drugs alone	- Short-term clinical cure rate improvement (RR 1.14, 95% CI 1.05–1.24) - Short-term mycological cure rate improvement (RR 1.06, 95% CI 1.02 to 1.10) - Reduction in first relapse rate at one month after treatment (RR 0.34, 95% CI 0.17–0.68) - No significant difference in long-term clinical cure rate at one month after treatment (RR 1.07, 95% CI 0.86–1.33) and at three months after treatment (RR 1.30, 95% CI 1.00–1.70) - No significant difference in long-term mycological cure rate at one month after treatment (RR 1.26, 95% CI 0.93–1.71) and at three months after treatment (RR 1.16, 95% CI 1.00–1.35)—No significant difference in serious adverse events (RR 0.80, 95% CI 0.22–2.94) - No significant difference in non-serious adverse events (RR 0.90, 95% CI 0.48–1.70)	High risk of bias; Very low to low confidence in estimate of effect (GRADE tool)	The results cannot be applied to pregnant women; women with rVVC, diabetes mellitus, or immunosuppressive disorders; or women taking immunosuppressant medication.
Hanson et al. [60]	2016	4 (2 Italy, 1 Sweden, 1 Nigeria)	2 Single Group studies, 2 RCT	155 in RCT, 88 in Single Group Studies	Combination probiotic product alone/ *L. rhamnosus* GR-1 and *L. reuteri* RC-14 and fluconazole/Combination probiotic product with fluconazole/Combination probiotics with metronidazole or clotrimazole	None of the studies demonstrated that the probiotic interventions were effective in the treatment of acute candidiasis. However, 2/4 found a significant reduction in recurrences in women who received the probiotic intervention. One found that probiotic bacteria were still detectable at 6 months in 9% of the intervention group.	2/4 High risk of bias, 4/4 poor quality	The rationale for dosing and/or the treatment duration were not provided in studies. The probiotic products’ specifics differed across the studies. The authors recommend doses between 10^9^ to 10^11^ CFUs by any route for probiotic interventions, although this stems from studies investigating BV
Ray et al. [62]	2011	1 study concerning Lactobacillus (USA)	RCT	114	3 arms of comparison; Clotrimazole, *Lactobacillus,* and Placebo	No definitive results in preventing an episode of VVC. Clotrimazole against lactobacillus (RR 1.11; 95% CI 0.45–2.76) and lactobacillus against placebo (RR 0.54; 95% CI 0.26–1.13).	Low quality (GRADE tool)	Findings apply to HIV-positive women
Abad et al. [59]	2009	4 studies reporting on VVC	2 RCTs, 1 Prospective crossover trial, 1 Quasi-experimental trial	399 in RCTs, 61 in others	Lactobacillus yogurt orally/Oral + Vaginal pessary *Lactobacillus*/*Lactobacillus* Vaginal suppository/Lactobacillus intravaginal capsule	1/2 studies showed a statistically significant decrease in risk of VVC (Study concerning yogurt administration) [RR 0.39 (CI 0.17–0.7); *p* < 0.009]. 2/2 studies reported non-significant results in treatment of VVC	No quality assessment	The studies were largely heterogenous, and no quality assessment was conducted
Van Kessel et al. [63]	2003	2 studies reporting on VVC	2 RCTs with crossover	47	oral yogurt with *L. acidophilus*	3-fold decrease in VVC in the intervention group in 1/2 studies	No quality assessment	The same study as in Abad et al. reported significant outcomes (only 13 patients completed the protocol)

VVC: Vulvovaginal Candidosis; RCT: Randomized Clinical Trials; RR: Risk Ratio; CI: Confidence Interval; GRADE: Grading of Recommendations, Assessment, Development, and Evaluation; CFU: Colony-Forming Units; OR: Odds Ratio; BV: Bacterial Vaginosis.

## 4. Discussion

VVC is a well-characterized mucosal infection that presents a significant clinical challenge primarily due to its recurrent nature and the pathogenic resilience of *Candida* species. Probiotics offer a promising adjunctive or alternative therapeutic strategy to antifungals in the management of VVC [33]. These beneficial microorganisms can be administered either orally or intravaginally. Oral administration facilitates colonization of the intestine, potentially influencing the vaginal flora via the gut–vaginal axis. Conversely, intravaginal delivery targets the site of infection directly, aiming at immediate colonization and action against *Candida* species. Similar to their use in BV [5], identification of proper population and timing remains the key to best utilizing their potential in women with acute or recurrent episodes.

### 4.1. Primary Treatment

Studies have shown variable results in the effectiveness of probiotics in the management of acute VVC infections [23,35,37,40,41,42,47,49]. Their use has beneficial effects in alleviating acute symptoms, including significant reduction of discharge and itching, enhancing patient comfort and quality of life [40]. In this context, Vahedpoor Z et al. [45] observed improvements in itching, discharge, and vaginal pH, suggesting that probiotics might help stabilize the vaginal environment and reduce inflammation. This comes in line with data demonstrating that probiotic vaginal administration could significantly increase Lactobacillus counts in vaginal samples [50]. We have recently shown that a vaginal probiotic gel with *L. plantarum* YUN-V2.0, *L. pentosus* YUN-V1.0, and *L. rhamnosus* YUN-S1.0 for 10 consecutive days showed a cure rate of 45%, which is below the expectations of a standard first-line treatment but still indicates that half of the patients were comfortable with the cream only, without the use of antifungals [37]. The systematic reviews and meta-analyses reviewed here provide a generally positive yet inconsistent view of the efficacy of probiotics in managing VVC [31,59,60,61,62,63,64]. While short-term benefits are frequently reported, the long-term efficacy remains mostly uncertain, as most of the pooled evidence shows sparse data regarding long-term clinical and mycological cure rates. However, probiotics can by no means substitute antifungal treatment in terms of quickly reducing the fungal load. The study by Mollazadeh-Narestan et al. [42] highlighted that, while probiotics showed some benefit, fluconazole had a significantly higher frequency of negative culture results after 60–65 days, suggesting that while probiotics may be useful in managing symptoms, they might not be as effective as conventional antifungal treatments in eradicating the yeast [42]. In contrast, Bertarello et al. [35] indicated that probiotics could achieve similar efficacy to standard treatments such as miconazole and provide significant symptom relief, suggesting a potential role for probiotics in managing VVC, particularly in terms of improving patient comfort and reducing symptoms such as discharge and itching.

### 4.2. Recurrence Prevention

When a cure does occur, maintenance therapy with probiotics could significantly reduce recurrence rates [55] and improve long-term clinical cure rates, highlighting the potential of probiotics in the ongoing management of rVVC [23,24,44]. Nonetheless, the identified studies demonstrate a diverse approach to probiotic applications, both in terms of the probiotic strains used and the formulations administered. Moreover, they vary in the specific outcomes measured, which presents significant challenges in judging the potential role of probiotics in preventing VVC [23,36,38,39,41,46,49]. For instance, the RCT by Pirotta et al. [56], which is the most powerful in this review, with 278 participants and a robust 2 × 2 factorial design, is particularly noteworthy in that it employed both an oral and a vaginal route of administration. It did not demonstrate a significant difference in the prevention of post-antibiotic vulvovaginitis between the probiotic and placebo groups. On the other hand, the multicenter RCT by Vladareanu et al. [58], which included 93 Caucasian women with a history of recurrent yeast vaginitis, observed significant improvements in vaginal health scores and reductions in symptoms such as vaginal mucosa redness and swelling following treatment with *L. plantarum* P17630. Similarly, in order to prevent recurrences, a 2014 observational study by Murina et al. [54] from Italy reported impressive results regarding the synergistic potential of combining vaginal probiotics with fluconazole to reduce recurrence rates. This study assessed a slow-release vaginal product containing *Lactobacillus* strains, following induction treatment with oral fluconazole, in 58 patients with acute symptomatic VVC who also had a history of rVVC, with four or more culture-confirmed episodes in a 12-month period. The investigated regimen reportedly resulted in 86.0% of the participants remaining free of clinical recurrence during the 10-week prophylactic phase and 85.7% remaining symptom-free over the subsequent 7-month observation period. Given the recurrent nature of VVC, more focus should be placed on investigating the efficacy of probiotics over more extended periods to fully understand their role in preventing relapses and sustaining vaginal health. Another limitation is the insufficient evidence regarding the ability of administered probiotic strains to effectively colonize the vagina. Successful colonization is critical for the prolonged efficacy of probiotics in modifying the vaginal microbiota and exerting their beneficial effects [65]. Without this sustained presence, the transient existence of probiotics may be inadequate to influence the underlying pathophysiology of VVC, particularly regarding long-term outcomes. Furthermore, the substantial heterogeneity among the included studies, as highlighted in several meta-analyses, complicates the aggregation of data and the interpretation of outcomes.

### 4.3. Adjuvant Therapy

Finally, the synergy between probiotics and antifungals may enhance the overall treatment efficacy. For instance, Martinez RC et al. [41] observed that the combination of fluconazole with probiotics resulted in higher cure rates of VVC compared to fluconazole alone (adjuvant probiotic therapy). Similarly, Pendharkar S et al. [43] demonstrated that adjunctive use of EcoVag^®^ with fluconazole not only achieved high cure rates at 6 months but also maintained these effects for up to 12 months, indicating sustained benefits when probiotics are used with antifungals.

### 4.4. Safety

Although relatively safe, there is always the potential risk that probiotics, as living organisms, could cause infections that require antibiotic treatment, particularly in individuals with underlying health conditions. Many probiotic strains are genetically modified in laboratories to enhance their health benefits. Therefore, the safety of each strain must be ensured and closely monitored to prevent environmental accumulation, possession of antibiotic selection markers, or transfer of harmful genetic information to other bacteria [2]. Safety and tolerability are consistently reported across the studies, including in our report, affirming that probiotics represent a low-risk intervention [52]. The absence of serious adverse effects and the general improvement in vaginal health parameters point to the potential benefits of probiotics in the management of VVC [53]. However, their overall effectiveness can be highly context-dependent, influenced by factors such as the probiotic strains used and possibly the route of administration. Despite promising results, the evidence remains fragmented due to methodological diversity and small sample sizes in many of the studies. The observational and retrospective studies, such as those by Reid et al. [57] and De Seta et al. [51], while providing valuable preliminary data, contribute low-quality evidence due to their inherent design limitations. Nonetheless, these studies support the safety and tolerability of probiotics, noting improvements in patient-reported outcomes and reductions in infection recurrence rates.

### 4.5. Cost–Effect Considerations

Despite the lack of robust clinical evidence, the demand for pro-/pre-biotic approaches to enhance vaginal health continues to grow, fueled by consumer interest and a multi-billion-USD global market. Probiotic cost-effectiveness has been demonstrated in the context of hospitalized adult patients receiving antibiotics, as well as the prevention of *Clostridium difficile*-associated diarrhea in children and adolescents [66,67]. In this case, probiotics have been found to be economically attractive across a wide range of plausible values, but not in all scenarios [66,67,68]. This was not the case though for the prevention of ventilator-associated pneumonia, where probiotics were found not to be cost-effective across wide ranges of plausible willingness-to-pay thresholds [69]. A paucity of cost-effectiveness data exists regarding its use in bacterial or fungal vaginosis, which, considering the lack of significant benefit despite their relative safety, does not support their use as standard-of-care in the management of VVC. Special focus should be put on how host factors, such as age, hormonal status (e.g., menopause), immune status, and underlying medical conditions (e.g., diabetes and HIV/AIDS), influence the effectiveness of probiotics in managing VVC. Understanding these factors can help tailor probiotic therapies to specific patient populations and optimize treatment outcomes and cost-effectiveness. This aspect could provide valuable insights into the personalized use of probiotics in clinical practice, addressing the variability in treatment response among individuals with different health profiles and resulting in patient risk stratification models that could maximize benefit–risk/cost ratios.

## 5. Conclusions

The diversity in study designs, outcome measures, and variations in probiotic formulations pose the main challenges in drawing definitive conclusions. The high risk of bias and overall low quality of evidence observed in many studies necessitate cautious interpretation of these results. As regulations surrounding probiotic product claims tighten [70], there is an urgent need for studies investigating standardized and well-defined probiotic products. High-quality, well-designed randomized controlled trials are then needed to establish the true efficacy and appropriate use of pro-/pre-biotics in the management of VVC, including well-defined endpoints, and investigations into the optimal duration, route, and timing of probiotic administration for maximum efficacy. We propose that future studies should study the benefit of probiotics in well-defined categories such as (1) acute probiotics treatment instead of antifungals, (2) adjuvant probiotic therapy together or after antifungals, and (3) VVC recurrence prevention using probiotics. Such research efforts are crucial to providing patients and healthcare providers with the evidence needed to make informed decisions about the potential benefits and limitations of incorporating pro-/pre-biotics into the clinical management of VVC, either as a standalone therapy/prophylaxis or as an adjunct to traditional antifungal treatments.
